# The importance of caveolin as a target in the prevention and treatment of diabetic cardiomyopathy

**DOI:** 10.3389/fimmu.2022.951381

**Published:** 2022-11-02

**Authors:** Weiyi Xia, Xia Li, Qingping Wu, Aimin Xu, Liangqing Zhang, Zhengyuan Xia

**Affiliations:** ^1^ Department of Anesthesiology, Affiliated Hospital of Guangdong Medical University, Guangdong, China; ^2^ Department of Orthopaedics and Traumatology, The University of Hong Kong, Hong Kong, Hong Kong SAR, China; ^3^ Department of Anesthesiology, Union Hospital, Tongji Medical College, Huazhong University of Science and Technology, Wuhan, China; ^4^ State Key Laboratory of Pharmaceutical Biotechnology, Department of Medicine, The University of Hong Kong, Hong Kong, Hong Kong SAR, China

**Keywords:** insulin signal pathway, caveolin 1, caveolin 3, diabetic cardiomyopathy, adiponectin, oxidative stress

## Abstract

The diabetic population has been increasing in the past decades and diabetic cardiomyopathy (DCM), a pathology that is defined by the presence of cardiac remodeling and dysfunction without conventional cardiac risk factors such as hypertension and coronary heart diseases, would eventually lead to fatal heart failure in the absence of effective treatment. Impaired insulin signaling, commonly known as insulin resistance, plays an important role in the development of DCM. A family of integral membrane proteins named caveolins (mainly caveolin-1 and caveolin-3 in the myocardium) and a protein hormone adiponectin (APN) have all been shown to be important for maintaining normal insulin signaling. Abnormalities in caveolins and APN have respectively been demonstrated to cause DCM. This review aims to summarize recent research findings of the roles and mechanisms of caveolins and APN in the development of DCM, and also explore the possible interplay between caveolins and APN.

## 1 Introduction

Diabetes is a well-recognized risk factor for cardiovascular disease, which is largely responsible for the significantly shorter life expectancy of people with diabetes as compared to those without diabetes (1, 2). As the prevalence of diabetes continues to rise, both clinical and experimental studies have demonstrated that the existence of diabetic cardiomyopathy (DCM) can cause significant changes in clinical presentation as well as in heart structure and function (3, 4). Persistent hyperglycemia adds an extra burden on the heart, and the severity of DCM progresses over time beginning early after diabetes onset, followed by ventricular dysfunction and ultimately life-threatening heart failure (5, 6). Although DCM develops during a long-term preclinical phase, diabetic patients with heart failure have substantially worse outcomes than non-diabetic patients, making the treatment of DCM a clinically pressing problem.

Integral membrane proteins caveolins 1 and 3 have long been demonstrated in preclinical studies to play critical roles in both diabetes and cardiovascular disease (7, 8). Caveolin-3 (Cav-3), the muscle-specific isoform of caveolin, has been proposed as a potential target for the prevention or treatment of DCM (9). Likewise, the reduction or impairment in the function of adiponectin (APN) has also been shown to be attributable to the development of DCM. However, the exact roles of the caveolins in the development of DCM are largely unclear, and the potential interactions between caveolins and APN in the progression of DCM has yet to be explored. Here, we summarize recent literature about the role of caveolins and APN in DCM, the relevant signaling pathways involved, and the potential interactions between caveolins and APN in the development and progression of DCM.

## 2 Overview of diabetic cardiomyopathy

### 2.1 Definition of diabetic cardiomyopathy

DCM, first proposed by Rubler et al. in 1972 (4), is currently defined as an abnormality of myocardial structure and function that occurs independently of cardiac risk factors such as hypertension or myocardial ischemia in diabetic patients. Despite the different etiologies underlying type 1 diabetes mellitus (T1DM) and type 2 diabetes mellitus (T2DM), the disorders of glucose metabolism and subsequent persistent hyperglycemia in both types of diabetes are closely related to the onset and development of DCM. T2DM, which is characterized by insulin resistance and consequent loss of normal glucose homeostasis, accounts for more than 90% of all diabetes patients, with a majority of them being adults and elders (10). Of every hundred elderly diabetics, approximately 12 develop heart failure and 6 are at risk of death (11), which is indicative of a strong association between T2DM and heart failure (12, 13). A recent population study results suggest that diabetes mellitus is an independent risk factor for the development of heart failure (HF) even for those without underlying diastolic dysfunction (14). This rationale is supported by the structural, functional and metabolic changes in diabetic cardiomyocytes in animal models (15–17).

While the understanding of diabetes-related cardiovascular complications has accumulated significantly over the past decades (18), definitive identification of DCM remains controversial. Most patients with T2DM suffer from other comorbidities, including obesity, hypertension and coronary heart disease, while persistent obesity itself is also a major risk factor for the development of other metabolic disorders and cardiovascular diseases (19, 20). Furthermore, symptoms of patients with chronic heart failure are nearly identical, regardless of the presence or absence of diabetes. Given such situations, it is challenging to identify whether DCM in diabetic patients is caused by insulin resistance or other existing comorbidities.

### 2.2 Clinical progression of diabetic cardiomyopathy

**Figure 1:**
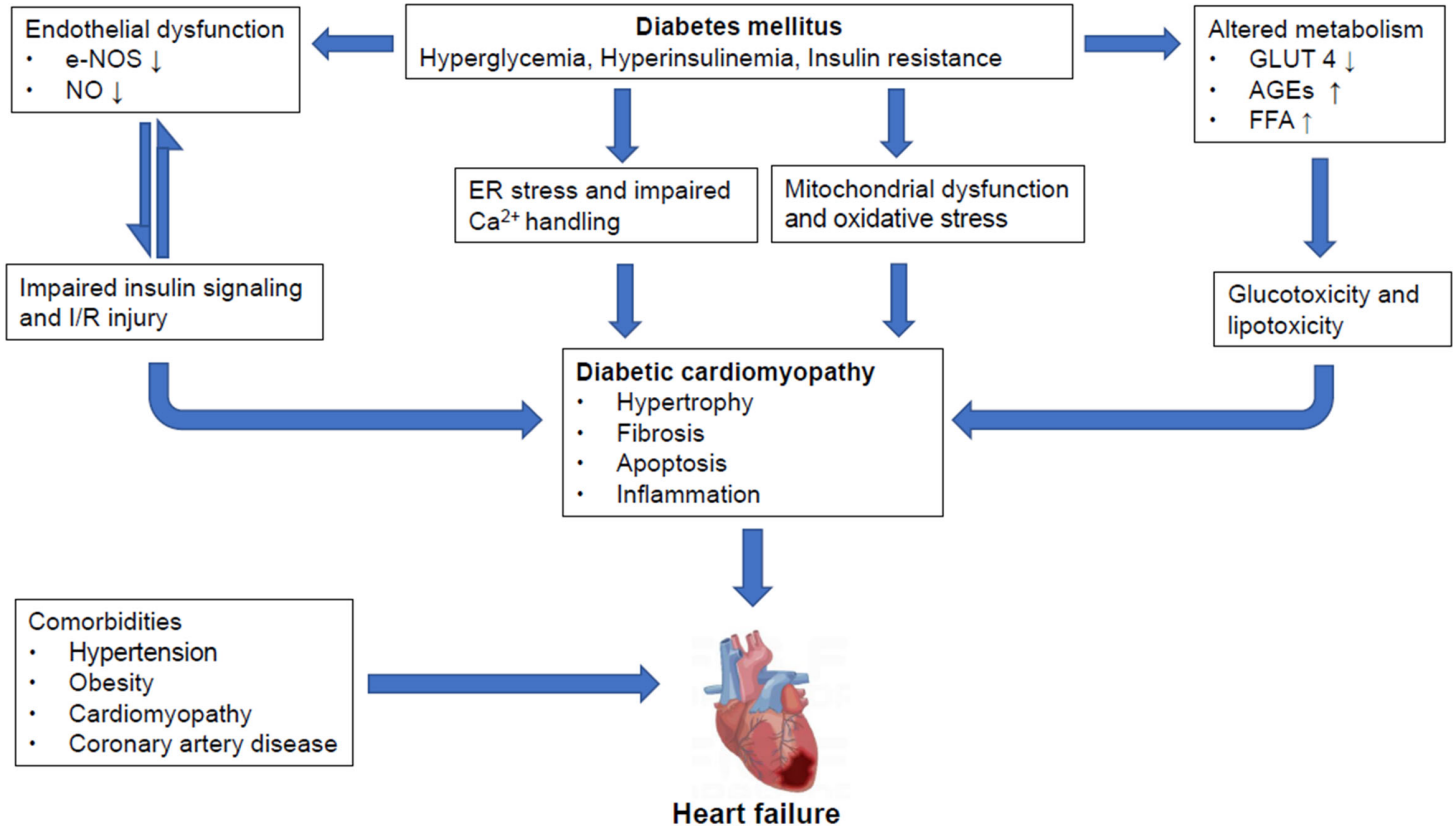
Overview of pathological mechanisms underlying diabetic cardiomyopathy and heart failure in diabetic mellitus Pathological stresses such as hyperglycemia, hyperinsulinemia and increased insulin resistance induce endothelial dysfunction through suppression of eNOS and nitric oxide (NO) production, causing impairments in insulin signaling which could further damage the integrity of endothelial tissues. And, reciprocal relationships exist between insulin resistance and endothelial dysfunction. Moreover, these instigators also alter metabolism by reducing GLUT4 expression, increasing AGEs and FFA production. All of which lead to glucotoxicity and lipotoxicity. Meanwhile, ER stress and impaired Ca2+ handling also occur, along with mitochondrial dysfunction and oxidative stress. These, together, cause myocardial hypertrophy, fibrosis, apoptosis and inflammation, contributing to the formation of diabetic cardiomyopathy (DCM) which may proceed to heart failure (HF). However, it is also important to point out that diabetic patients may often have other comorbidities including but not limited to hypertension, obesity, cardiomyopathy and coronary artery diseases that may result in HF. NO, nitrogen oxide; IR, ischemic reperfusion; AGEs, advanced end glycation products; ER, endoplasmic reticulum; DCM, diabetic cardiomyopathy; HF, heart failure.

Hearts of diabetic subjects present time-dependent structural, functional, and metabolic abnormalities, such as ventricular dysfunction, myocardial hypertrophy and fibrosis, cardiometabolic alterations, increased fat consumption and oxidative stress (21). However, the progression from DCM onset to end-stage heart failure is variable (**Figure 1**).

In the early stages of DCM, decreased left ventricular diastolic function usually presents as the first manifestation of myocardial involvement while the majority of diabetic patients remain asymptomatic (22, 23). Indeed, metabolic disorders that are associated with hyperglycemia precede the structural changes in DCM and are considered as contributors to the cardiogenic changes observed in diabetic patients. Under the condition of insulin resistance and hyperinsulinemia, the diabetic heart exhibits lower glucose uptake and decreased expression of glucose transporters-4 (GLUT-4) (24, 25), while the circulating free fatty acids (FFA) are increased as the main energy substrates (26, 27). On the macroscopic level, increased myocardial stiffness and atrial filling time are often observed with the presence of prolonged metabolic abnormalities (28). This hidden subclinical impairment in diastolic function could be detected in up to 75% of diabetic patients using tissue Doppler echocardiography (29, 30).

After the onset of DCM, the imbalance between substrate uptake and consumption results in myocardial hypertrophy followed by the excess accumulation of toxic intermediates (31) and dynamic changes at the cellular level (32). The increased formation of advanced glycation end products (AGEs), mitochondrial dysfunction, lipid accumulation, functional decline in calcium handling proteins and activated renin-angiotensin-aldosterone system (RAAS) have been known to accelerate the progression of DCM causing damage to the heart including increased fibrosis accompanied by decreased left ventricular compliance, impaired microvascular function and reduced ejection fraction during systole (33–35).

As DCM progresses, alterations in cardiac structures may become more pronounced and characterized by diffuse hypokinesis due to stretched and weakened heart muscles (36). In addition to structural changes in the heart, endothelial dysfunction is caused by biochemical effects such as reduced nitric oxide (NO) bioavailability, increased oxidative stress and inflammation in coronary vessels and capillaries (37). Due to long-term exposure to hyperglycemia, vascular homeostasis is broadly disrupted, leading to myocardial cell death and reduced blood flow and consequent myocardial ischemia along with myocardial fibrosis (38, 39). Some diabetic patients continue to develop heart failure with reduced ejection fraction (HFrEF), and the coexistence of these two conditions doubles the likelihood of a worse clinical outcome (40).

### 2.3 Molecular mechanisms and signaling pathways underlying diabetic cardiomyopathy

**Figure 2:**
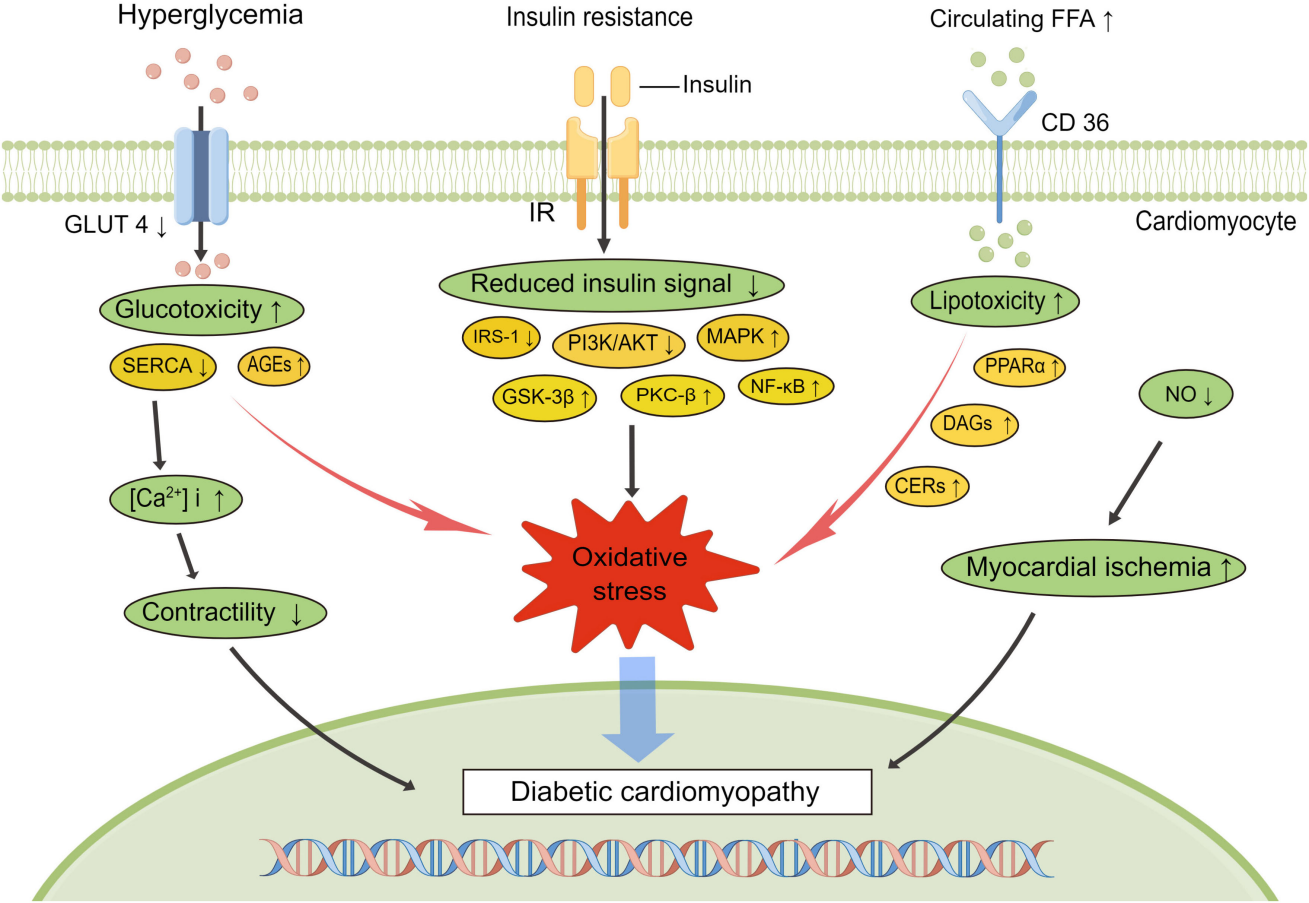
Signaling pathways involved in the development of diabetic cardiomyopathy. Diabetes is characterized by hyperglycemia, insulin resistance, and increased circulating free fatty acids, which lead to impaired diabetes signaling, increased inflammation, altered Ca^2+^ handling, and myocardial ischemia, exacerbating the progress of diabetic cardiomyopathy. AGEs, advanced end glycation products; SERCA, sarcoplasmic reticulum Ca^2+^-ATPase; NO, nitrogen oxide; IRS-1, insulin receptor substrate-1; PI3K, phosphoinositide 3-kinase; MAPK, mitogen-activated protein kinase; GSK-3β, glycogen synthase kinase-3β; PKC, protein kinase C; NF-κB, nuclear factor κB; PPARα, peroxisome proliferator-activated receptor alpha; DAGs, diacylglycerols; CERs, ceramides.

The regulation of glucose homeostasis is highly complex and integrates multiple signaling cascades and feedback loops. Hyperglycemia and insulin resistance are usually present prior to DCM onset and accompany DCM progression. It is well known that persistent hyperglycemia triggers a series of maladaptive processes in diabetic patients, such as insulin resistance, metabolic disturbances, oxidative stress and inflammation, which work together to promote the development of DCM (**Figure 2**). Diabetes presents a pro-inflammatory state, and oxidative stress–related signaling pathway impairment is proposed as a central event in the progression of DCM (41, 42).

Typically, FFA serves as the predominant energy source for the working heart, providing 50–70% of cardiac ATP demands *via* β-oxidation in the mitochondria (43). Peroxisome proliferator-activated receptor α (PPARα), an important transcriptional regulator, is mainly expressed in organs with high fatty acid (FA) oxidation rates and regulates the expression of genes relevant to lipid homeostasis (44). In the diabetic heart, PPARα is over activated by enhanced FA oxidation, which can subsequently result in the production of more reactive oxygen species (ROS) and mitochondrial uncoupling (45). Excessive ROS may be an important driver of diabetic myocardial inflammation. Elevated inflammatory cytokines (TNF-α, IL-6, IL-1β, TGF-β1), increase the expression of cell adhesion molecules like vascular cell adhesion molecule-1(VCAM-1) *via* nuclear factor κB (NF-κB) transcription factor activation associated with diastolic dysfunction which contribute to ventricular dysfunction in DCM (32). ROS generation also initiates the activation of other molecular stress signaling pathways, including mitogen-activated protein kinases (MAPK), extracellular signal-regulated kinases 1 and 2 (ERK1/2) and Jun N-terminal kinases (JUNK) (46, 47). Lipotoxicity arises when FA uptake exceeds mitochondrial oxidative capacity, and the excess storage of toxic lipid metabolites, such as diacylglycerols (DAGs) and ceramides (CERs), further impairing insulin metabolic signaling (48, 49). Protein kinase C (PKC), activated predominately by the intermediate DAG, is associated with a range of vascular abnormalities including endothelial dysfunction, changes of vascular permeability and impaired angiogenesis (50), and the β-isoform of PKC (PKCβ) is most directly related to vascular dysfunction and cardiac hypertrophy (51–53). Meanwhile, hyperglycemia-induced endoplasmic reticulum (ER) stress disrupts the structural integrity of ER and leads to cell death *via* a pathway known as the unfolded protein response (UPR) (54). Another consequence of ER stress is calcium imbalance within the diabetic hearts due to abnormal calcium release from sarcoplasmic reticulum and decreased activity of sarcoplasmic reticulum Ca^2+^-ATPase (SERCA) (55, 56). Increase in ROS production, calcium overload, and changes in mitochondrial permeability transition pore (mPTP) collectively promote cardiomyocyte apoptosis. Owing to the impaired antioxidant preservation of the diabetic myocardium, endothelial nitric oxide synthase (eNOS) activity and NO bioavailability are reduced, resulting in vascular endothelial dysfunction, which is mainly manifested by decreased vascular reactivity and myocardial blood flow (57–59). In addition to the direct damage to blood vessels, the increase in AGEs secondary to hyperglycemia can also promote the generation of oxidative stress by binding to the primary cell-surface receptors (RAGE) (60). These processes may activate the expression of NF-κB, leading to long-term exposure of myocardium to chronic inflammation (61, 62).

### 2.4 The increased susceptibility to ischemia/reperfusion injury in diabetes

Metabolic disturbances and aberrant expression of signaling pathways in diabetics greatly impair normal cardiac function and also makes the heart more vulnerable to myocardial ischemia/reperfusion(I/R) injury. Clinically, vascular damage caused by increased oxidative stress increases the risk of cardiovascular complications in diabetic patients compared with the general population, such as coronary heart disease and myocardial infarction (63). During the progression of DCM, myocardial ischemia at the microvascular and macrovascular levels has direct adverse effects on cardiac function which exacerbates the progression of DCM to heart failure.

Microvascular dysfunction in patients with diabetes is a multifactorial systemic phenomenon. Animal models of diabetes displayed the accelerated formation of atherosclerosis (64), reduced angiogenic response and myocardial endothelial cell density, which were functionally manifested as impaired collateral dependent perfusion and left ventricular function (65). The increase in collagen deposition observed in diabetic mice (66, 67) may contribute to the thickening of the basement membrane of capillaries and reduction in diameter, leading to impaired myocardial perfusion, which in turn exacerbates myocardial remodeling and fibrosis. In addition, increased oxidative stress induced by hyperglycemia can directly lead to cardiomyocyte death (68, 69). It is well known that NO plays a central role in maintaining cardiovascular homeostasis. Studies have shown that enhancing NO levels can effectively attenuate tissue ischemia by promoting vascular remodeling under the condition of metabolic dysfunction induced by high glucose (70). However, metabolic disorders inherent in diabetes, including hyperglycemia, insulin resistance, and increased FFA levels result in endothelial dysfunction and impaired angiogenic response to ischemia, ultimately leading to acute coronary events and heart failure (71). Collectively, the susceptibility of diabetic hearts to I/R injury is closely related to the duration and severity of hyperglycemia (72), and diabetes combined with ischemic cardiomyopathy may together worsen global ventricular mechanics and dysfunction.

## 3 Caveolin and its potential as a treatment target for diabetic cardiomyopathy

### 3.1 Caveolins in the heart

Caveolae, flask-shaped invaginations of the plasma membrane first identified in 1953 by electron microscopy (73), are specialized forms of lipid rafts. After decades of research, it is known that caveolae act not only in cholesterol transport, endocytosis and transcytosis, but also as cellular signaling hubs regulated by proteins of the caveolin family (74). Caveolins mainly exist in three forms that are derived from distinct genes: caveolin-1 (Cav-1), caveolin-2 (Cav-2) and caveolin-3 (Cav-3), all of which are key structural proteins essential for caveolae formation (75). Cav-1 and Cav-2 are generally expressed together in cells other than striated muscle, such as endothelial cells, adipocytes and fibroblasts (76), whereas Cav-3 is muscle-specific, predominantly in skeletal and cardiac muscles and smooth muscle (77). Interestingly, Cav-2 must interact with Cav-1 to be stable and acts as an accessory protein of Cav-1, target to the plasma membrane where it binds to cholesterol (78). Since Cav-2 will not exist independently in the absence of Cav-1, we focus on Cav-1 and Cav-3 here. Of note, as stated in the European Society of Cardiology Guidelines on diabetes, pre-diabetes and cardiovascular diseases (79), empagliflozin, among others, has been newly recommended as one of the first line options for glucose lowing treatment, while its protective mechanism involves Cav-1 signaling (80). This signifies the importance of caveolins in the treatment of diabetic complications including diabetic cardiomyopathy. While both Cav-1 and Cav-3 are expressed in cardiac myocytes, available evidence support that Cav-3 is most abundant in cardiomyocytes and Cav-1 is more abundant in vascular endothelial cells. The deficiency in either Cav-1 or Cav-3 can lead to DCM, but their exact signaling pathway may vary at different stages of diabetes, despite that complex interplay among Cav-1, Cav-3, eNOS and APN exist physiologically or pathologically in the case of DCM development.

### 3.2 Caveolin-regulated signaling in the heart and its interplay with eNOS

In the heart, caveolins are highly expressed in myocytes, fibroblasts, vascular endothelial cells and smooth muscle cells, and the knockout (KO) of different types of caveolins results in different cardiovascular phenotypes (**Table 1**). It is widely accepted that Cav-3 is dominant in cardiomyocytes, and the loss of Cav-3 induces cardiac hypertrophy and cardiomyopathy *via* p42/44 MAPK pathways (85). Moreover, Cav-3 is involved in the formation of T-tubules (98), which are essential for regulating calcium homeostasis and excitation-contraction (EC) coupling in ventricular cardiomyocytes (99). Hearts from Cav-3 KO mice show decreased t-tubular Ca^2+^ current (*I*_Ca_) density *via* L-type Ca^2+^ channels (LTCCs), causing cellular hypertrophy and altered EC coupling (86–88). Cav-1 has also been shown to be associated with the left ventricular hypertrophy and pulmonary hypertension, which in turn leads to right ventricular dilation (81). Due to the negative regulation of p42/44 MAPK pathways by Cav-1 (100), the histological changes within the heart of Cav-1 KO mice are associated with the hyperactivation of the p42/44 MAPK cascade in cardiac fibroblasts (82). NO production mediated by eNOS exerts an important protective effect on endothelial cells, and the expression and activity of eNOS are negatively regulated by the direct protein-protein interaction with Cav-1 (101, 102).

**Table 1:** Summary of pathological findings in Cav-1/Cav-3 KO mice.

	Cav-1 KO mice	Cav-3 KO mice
Cardiomyocytes	Hypertrophy ↑ (81, 82) Insulin receptor↓ (83, 84)	Hypertrophy ↑ (85) MAPK pathway ↑ (85) *I*_Ca_ density ↓ (86–88) Insulin resistance ↑ (89)
Fibroblasts	MAPK pathway ↑ (82)	–
Endothelial cells	Pulmonary hypertension ↑ (90, 91) eNOS activity ↑ (92–94) NO production ↑ (92–94) Vasodilatation ↑ (92–94) Contractility ↓ (92–94) vascular permeability ↑ (95, 96) atherosclerosis susceptibility ↓ (97)	–
vascular smooth muscle	Contractility ↓ (92–94)	–

Cav, Caveolin; KO, knockout; MAPK - mitogen-activated protein kinases; I _Ca,_ t-tubular Ca^2+^ current; eNOS endothelial nitric oxide synthase.

The arrow “↑” indicates up-regulation or increase and “↓” represents down-regulation or decrease.

In addition, hyperactivity of eNOS in Cav-1 deficient mice has been shown to induce the production of NO, leading to continued vasodilation and decreased contractility (92–94), as well as elevated vascular permeability (95, 96). However, the lack of Cav-1 in endothelial cells was found to reduce susceptibility to atherosclerosis in Cav-1 and atherosclerosis-prone apolipoprotein E (ApoE) double KO mice (97). Recent studies have shown that Cav-1 deficiency is associated with increased pulmonary arterial pressure and arterial stiffness (90, 91).

In the context of DCM, an early study by Esberg and Ren demonstrated that abnormal eNOS signaling is a critical player leading to the development of DCM (103). Ensuing studies by us (104, 105) and others (106) further demonstrated that impairment in Cav-3/eNOS complex formation represents a key mechanism of DCM, and the impairment in cardiac Cav-3/eNOS signaling (104) or reduction of cardiac adiponectin expression (107) in diabetic rats could be restored by antioxidant treatment with N-acetylcysteine (104). In addition, APN KO or adiponectin receptor 1(AdipoR1) knock-down impaired cardiac Cav-3 signaling in diabetic mice that could be restored by APN supplementation in the early stage of diabetes (108). Furthermore, the expression of Cav-1 and eNOS are reduced significantly in endothelial cells that were taken from human muscle biopsies in subjects with type 2 diabetes (109). This alters the interaction between Cav-1 and eNOS, suggesting that a complex interplay of APN, eNOS, Cav-1 and 3 may collectively regulate the development and progression of DCM.

### 3.3 Role of caveolae and caveolins in insulin signaling

Insulin resistance is the major metabolic abnormality that contributes to DCM, which is characterized by reduced tissue response to insulin due to impaired insulin signaling pathways. At the cellular level, the binding of insulin to the insulin receptor (IR), a transmembrane receptor tyrosine kinase that exists as a heterotetramer with two extracellular α-subunits that contain the insulin-binding sites and two transmembrane β-subunits with the kinase domain (110), activates downstream signaling. Insulin receptor substrate-1 (IRS-1), a primary downstream mediator of IR signaling, was found in the plasma membrane of adipocytes and colocalized with the IR in caveolae (111). In the caveolin-enriched fraction, the levels of insulin-induced tyrosine phosphorylation of IRS-1 were significantly higher than other sites, indicating that IR in caveolae plays a crucial role in initiating insulin signaling pathways (112). Cholesterol depletion using methyl-β-cyclodextrin in adipocytes not only destroyed caveolae structures but also concomitantly attenuated insulin-stimulated glucose transport and insulin signal transduction pathways (113–115).

Cav-1 contains a scaffolding domain (SD) which is the principal structural component associated with the regulation of IR-activated signal transduction that originates in caveolae which are present at high levels in adipocytes. In fact, functional insulin signaling is highly dependent on Cav-1. Experiments *in vitro* showed that the scaffolding domain of Cav-1 can interact with the kinase domain of β-subunits on IR and enhance the IRS-1 phosphorylation that activates downstream signaling pathways (116, 117). Cav-1 KO mice showed significant insulin resistance and lower levels of IR, which may be associated with accelerated degradation of IR (83, 84). Cav-3 gene mutations have also been found to lead to impaired insulin stimulated glucose metabolism in myocytes (118, 119). It has long been known that in response to insulin, translocation of GLUT4 to the plasma membrane of adipocytes is a prerequisite for glucose transport (120). Previous studies revealed the morphological localization of GLUT4 in caveolae after insulin stimulation (121), which coincided with the increased levels of glucose uptake (122). All of the above studies have demonstrated that the caveolae environment plays an important role in the insulin signaling pathways.

It should be noted, however, with regards to the role of caveolins in signal transduction, the concept of an interaction between the caveolin scaffolding domain and a ‘caveolin binding motif’ in associated signalling proteins has been questioned for structural/biophysical reasons because the scaffolding region interacts with cholesterol and is in close proximity to the membrane and thus it might even be buried in the outer leaflet of the membrane as suspected by some researchers (123, 124). Thus, the nature of the interaction between caveolins and signalling proteins at the inner plasma membrane of caveolae remain unclear, although most caveolar-associated proteins are thought to localize there based on their lipophilicity.

However, a more recent study showed that there exists a strong eNOS co-localization with the Eps15 homology domain containing protein 2 (EHD2) signal in mouse aorta cryostat sections (125). Meanwhile, Cav-1 and EHD2 showed a striking co-localization in cryostat sections of small mesenteric arteries and also in human umbilical vein endothelial cells (125). These findings suggest the possibility that Cav-1 may bind or interact with eNOS *via* EHD2. Furthermore, EHD2 has been shown to localize to the caveolar neck region (126) and confine caveolae to the plasma membrane through association with actin (127). EHD2 may also undergo a series of conformational changes to align its phospholipid binding sites with the membrane and facilitate oligomerization of EHD2 into ring-like structures (128–130). Presumably, EHD2 may have an impact on the caveolin scaffolding domain conformationally and/or functionally. Study has shown that cavin3 interacts with Cav-1 *via* cavin1 and increases surface dynamics of caveolae. Cavin3 and EDH2, which promote release and constrain caveolae at the membrane respectively, have also demonstrated a regulation role in maintaining the equilibrium between surface-connected and surface-dissociated caveolae (131). EHD2 has been shown to regulate cellular fatty acid uptake *via* its impact on caveolar dynamics and insulin signaling (132). In addition, cavin2, which is also one of the caveolae-related proteins, has been shown to enhance the stability of insulin receptor and regulate insulin signaling through direct association at the plasma membrane in adipocytes (133), and cavin2 KO in mice not only resulted in increased insulin resistance but was also associated a decrease in the density of caveolae in the epididymal white adipose tissue (133). On the other hand, cavin4 KO in mice has been shown to protect against myocardial ischemia reperfusion injury (134), but whether or how caveolae is involved in cavin4 KO mediated cardioprotection is unclear since cavin4 KO did not cause a reduction or deformation of caveolae (135).

### 3.4 Caveolins as potential therapeutic targets for diabetic cardiomyopathy

Further understanding of the central role of caveolins in cardiac and insulin signaling under normal conditions will facilitate discovery of new treatment approaches for DCM. In insulin signaling cascades, phosphatidylinositol 3-kinase (PI3K) activates the major downstream effector Akt kinase, also termed protein kinase B (PKB), which modulates glucose uptake by facilitating GLUT4 translocation (136). Short-term exposure of mature adipocytes to glucose reduces Cav-1 expression and insulin sensitivity, but during adipocyte differentiation, chronic exposure to high glucose triggers adaptive response by adipocytes, manifested by increased protein expression of Cav-1, IR, and Akt (137, 138). However, under the influence of obesity-related risk factors, insulin sensitivity still decreases and eventually insulin resistance develops (139). The latest study results suggested that insulin treatment significantly upregulated the PI3K/Akt signaling pathway and insulin sensitivity in diabetic mice, while Cav-1 gene silencing eliminated this effect (140). Consistently, increased expression of Cav-1 in skeletal muscle can also improve insulin sensitivity (141), which may be related to the positive regulation of IRα and IRS-1 levels by Cav-1 (142). Since skeletal muscle is a major target organ for insulin‐mediated glucose utilization, functional analyses of Cav-3 KO mice indicated that lack of Cav-3 caused insulin resistance that was associated with reduced IRS-1 and Akt levels (89). Overexpression of Cav-3 increased the activity of IR and promoted the phosphorylation of IRS-1 without affecting expression of IR (117). Interestingly, another study showed that Cav-3 activated the Akt signaling pathway and enhanced GLUT4 translocation and glucose uptake, independent of insulin stimulation (143). In cardiomyocytes, Cav-3 and IR directly interacted to regulate glucose uptake (144). Insulin resistance attenuated the translocation of Akt and Cav-3 to the caveolae, and activation of Akt contributed to the uptake of glucose by cardiomyocytes (145). These findings suggest that caveolins have not only a structural role but also act as an enhancer in insulin signaling transduction pathways.

eNOS is expressed mainly in vascular endothelial cells and also in cardiomyocytes and other muscle cells, albeit at significantly lower levels. Previous studies have shown that the presence of Cav-1 is necessary for insulin-stimulated eNOS phosphorylation (146). If Cav-1 is knocked out, the endothelial-dependent vascular response mediated by eNOS lacks physiological regulation (93). Intermittent hypoxia decreased NO production in coronary endothelial cells but upregulated Cav-1 expression, which in turn impaired insulin-dependent Akt and eNOS activity (147). The loss of eNOS activity is a common and early pathological change in diabetic patients, and thus promoting Cav-1 expression in endothelial cells could improve insulin signaling and glycemic control. And, reciprocal relationships exist between insulin resistance and endothelial dysfunction (148) (**Figure 1**). In addition to NO, endothelium-derived hyperpolarizing factor (EDHF) also plays an important role in regulating vascular tone in small resistance vessels such as coronary microvessels (149, 150). NO-mediated coronary vasodilation was impaired in diabetes, and compensatory interaction of EDHF with Cav-1 maintained vasodilation during diabetic myocardial ischemia (151).

The cardioprotective effect of caveolins against I/R injury has also been observed *in vitro* and *in vivo*. Cav-1 KO mice exhibited more severe left ventricular dysfunction and lower survival after myocardial infarction compared with controls (152). Overexpression of the muscle-specific Cav-3 enhances myocardial tolerance to ischemia by increasing the expression of glycogen synthase kinase-3β (GSK-3β), natriuretic peptide and Akt phosphorylation (153). Akt activation has been reported to regulate GSK-3β-mediated mPTP opening to prevent myocardial I/R injury (154). Impaired mitochondrial function associated with mPTP opening during I/R injury has also been implicated in the progression of DCM. Tocotrienol antioxidant can differentially regulate the binding of Cav-1 and Cav-3 to p38 MAPK, increasing the levels of pro-survival signals like eNOS and heme oxygenase (HO-1) to protect the heart from ischemic injury (155). Moreover, Lei et al. (105) found that restoring Cav-3 expression in diabetic myocardium can inhibit the overactivation of PKCβ and alleviate diastolic dysfunction by rescuing Akt-eNOS-NO signaling. Subsequently, the authors confirmed that enhancing the binding of Cav-3 to eNOS in diabetic rats by antioxidants could attenuate myocardial dysfunction and I/R injury (104). Sun et al. (106) revealed that the interaction between NO and H_2_S reduced hyperglycemia-induced ROS generation, cardiomyocyte apoptosis and hypertrophy by activation of the Cav-3/eNOS complex, providing a novel target to treat DCM. A more recent study further showed that Cav-3 overexpression protects diabetic hearts from acute myocardial infarction/reperfusion injury through activating the adrenoceptor β2 (ADRB2) and cAMP/PKA signaling pathways (156). Treatment that can reverse hyperglycemia-induced impairment in Cav-1 expression attenuates DCM (157), while pharmacological inhibition of the guanosine triphosphate cyclohydrolase 1 (GCH1), the rate-limiting enzyme in *de novo* synthesis of the eNOS cofactor tetrahydrobiopterin (BH4), has been shown to enhance tyrosine phosphorylation of Cav-1 and subsequently faciliates the development of DCM (158). These findings collectively signify the importance to target Cav-1 and Cav-3 in the treatment of DCM.

### 3.5 Interplay between Caveolins and adiponectin signaling

Myocardial ischemic injury is usually accompanied by tissue hypoxia. Hypoxia in mature adipocytes resulted in a marked reduction of important mediators in the insulin signaling pathway, such as Akt and IR phosphorylation (159). Furthermore, the level of Cav-1 was also downregulated, which could be explained by the possible destruction of the caveolae structure during hypoxia (159). Elevated levels of proinflammatory cytokine IL-6 and decreased levels of APN in hypoxic adipose tissue are also one of the mechanisms contributing to decreased insulin sensitivity (160, 161). These results illustrated the regulatory roles of caveolin and APN in the insulin signaling pathway. Indeed, Cav-1 KO has been shown to moderately but significantly reduce plasma levels of adiponectin in 3 month-old mice (162) and thus potentially impairs APN signalling. Furthermore, the fact that AdipoR1 and Cav-1 colocalized and coprecipitated in human umbilical vein endothelial cells and that APN mediated inhibition of TNF-α induced inflammation needed the participation of Cav-1 to potentiate AdipoR1 activation (163) supports a tight interaction in between Cav-1 and APN. These findings suggest that it is possible that reconstitution of Cav-1 in Cav-1 global KO mouse should restore APN signaling in cardiomyocytes, although related studies have not been reported to our knowledge. Additionally, either Cav-1 KO or Cav-3 KO significantly enhanced traumatic brain injury induced neuroinflammation (164), highlighting the importance of Cav-1 and Cav-3 in organ protection in pathological conditions. Likewise, a most recent study also reported a significant role of APN/AdiopR1 signaling in protecting against traumatic brain injury (165), nevertheless a potential of interplay between caveolin and APN signaling in this pathology has yet to be explored. The recent finding that APN secretion by exocytosis in white adipocytes is Cav-1-dependent (166) suggests that caveolin may affect APN signaling *via* diverse mechanisms.

## 4 Adiponectin and its potential as a treatment target for diabetic cardiomyopathy

### 4.1 Adiponectin and its receptors

In the past two decades, protective effects of APN against pathological events were observed in various cells, including suppressing cell death, inhibiting inflammation and enhancing cell survival. Understanding the structures of both APN and its receptors will likely provide critical insights toward a better understanding of the molecular mechanisms.

APN was first identified in 1995 (167) and was known to be specifically expressed in adipose tissue and fully differentiated adipocytes (168). While adipokines like omentin and chemerin are also cardioprotective, APN is the most abundant adipokines in adipose tissue, and possesses insulin-sensitizing effects (169). Through both size fractionation analysis and chemical crosslinking assays, Wang and his colleagues (170) found that APN may have multiple complexes and biochemical properties of APN may vary depending on the combination of its subunits. Similar results were shown again by Hu et al. (169) using mouse APN in which the study provided evidence that APN exists as multiple complexes with different molecular weights (171). These studies also suggested that APN plays a role in regulating whole body energy homeostasis.

Overall, three complexes - low molecular form (LMW), middle molecular form (MMW) and high molecular form (HMW) of APN were discovered. Along with the total level of APN, different distributions of the complexes may contribute to distinct downstream biological effects (170). However, it is the ratio of HMW to the total level of APN that plays a major role in determining the insulin sensitivity in both rodent and human studies. Apart from the molecule itself, APN signaling is also worth attention. APN signaling is mediated *via* two main APN receptors (AdipoRs): adiponectin receptor 1 (AdipoR1) and adiponectin receptor 2 (AdipoR2) (172). Findings from multiple *in vitro* studies involving glucose production assays (173) and *in vivo* studies in various rodent diabetic models (171–173) revealed that both AdipoR1 and AdipoR2 can mediate actions that are exerted through adenosine monophosphate-activated protein kinase (AMPK) signaling, a well-known metabolic pathway in the body. Moreover, early cloning studies in murine models identified that AdipoR1 is ubiquitously expressed, but most abundant in skeletal muscle, while AdipoR2 is mainly expressed in the liver. Expression of AdipoR1 in ventricular cardiomyocytes is estimated to be approximately 50% of the levels observed in skeletal muscle, while AdipoR2 expression is similar between the liver and cardiomyocytes (174).

Following the discovery of AdipoR1 and AdipoR2, Hu et al. (169) identified T-cadherin, a third APN receptor that specifically binds to MMW and HMW APN. A few years later, Denzel (175) found that T-cadherin is expressed in cardiac myocytes and mediates the antihypertrophic role of APN. However, this molecule lacks an intracellular domain, and thus cannot induce downstream signaling independently. While the functional role of T-cadherin in APN signaling is controversial, studies using T-cadherin KO mice and rats indicate that T-cadherin has a vital role in assembling APN near AdipoR1 and AdipoR2 to facilitate their signaling. In addition, deficiency of T-cadherin has been shown to increase infarct size induced by ischemia/reperfusion and reduce induction of AMPK (175). Studies have also been carried out to assess the binding affinities of various forms of APN to the AdipoRs, where it has been demonstrated that AdipoR1 preferentially binds globular APN, while AdipoR2 and T-cadherin binds HMW adiponectin (169, 176).

Both human and murine cardiomyocyte-derived APN has been demonstrated to be biologically active, protecting cardiomyocytes from simulated ischemia/reperfusion injury and directly impacting cardiac metabolism by activating APN receptors *via* an autocrine/paracrine mechanism (177, 178). The epicardial and pericardial adipose tissue were also found to secret proinflammatory adipocytokines that may exert local paracrine effects on the heart (168). A subsequent study from Achari and Jain (179) found that HMW and globular adiponectin (a proteolytic cleavage product of full length APN) are the most biologically active forms of APN thought to be primarily responsible for mediating its cardioprotective effects. In fact, people with APN gene defects are susceptible to LV hypertrophy and diastolic dysfunction (180).

### 4.2 Adiponectin as potential treatment targets for diabetic cardiomyopathy and myocardial ischemia/reperfusion injury in diabetes

It is well-understood that pathologies in DCM contribute to left ventricular diastolic dysfunction, resulting in increased LV end-diastolic pressure caused by impaired LV filling, reduced elasticity, and increased relaxation time and ultimately leads to myocardial ischemia and HF (181). Although various treatments are available for HF, none of them are specific to treating patients whose HF is caused by diabetes. Due to the ability of metformin to reduce gluconeogenesis and glycogenolysis in the liver, to increase glucose uptake in skeletal muscle and thus reduce FFA induced AMPK signaling (182), metformin is often used as the first-line pharmacotherapy for glycemic control among all medications (183). However, metformin is contraindicated in HF patients due to its side effect of lactic acidosis. Furthermore, the issue with DCM is the extent to which cellular damage and pathology that is confined to the heart. In this case, traditional non-specific pharmacotherapies such as metformin may not be effective since their secondary off-target effects are not specific (168). To the extent glucose-lowering agents are effective, they do not alleviate the underlying pathologies of DCM. This highlights the need to identify novel therapeutic targets and/or develop new treatments for this condition.

Since its first discovery in 1995, both clinical and experimental studies have pointed to the function of APN and its receptors in preventing cardiovascular dysfunction and remodeling of the heart (184–186). It was revealed that the heart has a local APN signaling system, which is downregulated in the diabetic heart of animal models and in patients. Moreover, expression levels of both the APN and its receptor are reduced in patients with T2DM (187). In cardiomyocytes, anti-inflammatory, anti-fibrotic and anti-apoptotic mechanisms could all be induced by APN (188). Clinical studies suggest that plasma APN levels are lower (6 fold) in diabetic subjects with cardiac comorbidities than in non-diabetic subjects, suggesting APN may be a predictor of cardiovascular risk in diabetes (189). On the other hand, experimental studies provided evidence that APN deficiency promotes cardiac hypertrophy, fibrosis and remodeling, which are all key contributors to diastolic dysfunction by promoting LV wall stiffening (190). Furthermore, recombinant globular APN treatment (2.5 ug/ml) ameliorates hypertrophy and fibrosis *via* activation of AdipoR1, APPL1 and AMPK in angiotensin II-treated rat atrial myocytes and fibroblasts (191). An increase in myocardial oxygen consumption which reduces mitochondrial oxidative phosphorylation and cardiac efficiency was observed in AdipoR1 KO mice (192). Conversely, mice overexpressing AdipoR1 exhibit reduced cardiac lipids, oxidative stress and metabolic dysfunction (193). Apart from increasing AdipoR1 expression, reducing ERK activation and hypertrophy *via* AMPK (194, 195), cardioprotective effects of APN are further demonstrated in cardiomyocytes through reduction of cardiac fibrosis (196, 197).

### 4.3 Possible essential role of caveolins in APN cardioprotection against diabetic cardiomyopathy or myocardial ischemia/reperfusion injury

#### 4.3.1 Role of caveolins in APN cardioprotection against myocardial ischemia/reperfusion injury

The role Cav-3 plays in APN transmembrane signaling and APN-induced anti-ischemic/cardioprotective actions were first demonstrated in the study of Ma and colleagues in 2012 (198). Since it has been well-established that ischemic heart disease is the main cause of death in patients with diabetes, we believe that understanding and defining the molecular basis of Cav-3 and APN signaling may help identifying novel therapeutic targets given that changes in either Cav-3 or APN are tightly associated with diabetes and ischemic heart disease. Restoration of Cav-3 and/or APN signaling may not only reduce the risk of myocardial ischemia, but also decrease cardiovascular mortality in diabetic patients. Within the categories of ischemic heart diseases, MI is a major perioperative complication in patients with diabetes (198). Although reperfusion therapies could restore coronary blood flow, lethal tissue injury known as “reperfusion injury” may also occur. Ischemic postconditioning (IPo) is a phenomenon in which brief repetitive episodes of ischemia and reperfusion immediately following the onset of reperfusion can protect the hearts against myocardial infarction reperfusion injury (MIRI) by restoring the impaired AdipoR1/Caveolin-3 signaling under diabetic condition (108). In addition, the cardioprotective effect of APN against myocardial ischemia reperfusion injury seen in wild-type mice has been shown to be significantly compromised or completely lost in mice with Cav-3 knock out, despite the expression level of key APN signaling molecules was normal in Cav-3 KO mice (198). Furthermore, APN receptor 1 (AdipoR1) has been shown to colocalize with Cav-3 in the heart, forming AdipoR1/Cav-3 complex (198). However, the potential interplay between Cav-3 and APN in the context of diabetic cardiomyopathy has yet to be explored.

#### 4.3.2 Role of caveolins in APN cardioprotection against myocardial ischemia/reperfusion injury in functionally impaired diabetic hearts

APN interacts with both Cav-3 and Cav-1 *via* different pathways to protect against MIRI by reducing myocardial oxidative/nitrate stress and activating endothelial nitric oxide synthase (eNOS), thereby increasing NO bioavailability (199). Although both Cav-1 KO and Cav-3 KO mice exhibited reduced insulin response in Comb’s study (200), only Cav-3 KO mice exhibit typical type-2 diabetic changes, including increased adiposity, decreased glucose uptake, reduced skeletal muscle glucose metabolic flux, and increased plasma leptin levels. Importantly, older Cav-3 null mice develop pathologic cardiac phenotypes. However, Cav-3 KO mice at age 2 months do not exhibit any myopathic changes (200), further demonstrating the role of APN/Cav-3 signaling in the progression of diabetes. A similar result was seen in Li’s study (108) with 4 week and 8 week old diabetic mice whose cardiac functions were impaired. In their study (108), it was found that the reason why diabetic hearts lose responsiveness to IPo cardioprotection was mainly due to reduction of APN in the early stage of diabetes, whereas the progression of diabetes caused the impairment of AdipoR1/Cav-3 signaling, and in the late case, APN supplementation in combination with IPo could not restore diabetic heart responsiveness (198). This being the case, further studies should focus on the Cav-3 and AdipoR1 signaling pathway, in particular their interplay, which could be a possible therapeutic target.

NO, which is increased through AdipoR1/Cav-1 signaling in cardiomyocytes, represents one of the most important defense mechanisms against MIRI and is also one of the major mediators of IPo cardioprotection (201). In the same study (108), IPo conferred cardioprotective effects through APN that were associated with reduced postischemic nitrotyrosine formation and increased cardiac NO production in WT but not in Adipo -/- mice. These data suggest that IPo confers cardioprotection by reducing myocardial oxidative stress and increasing NO bioavailability through APN (198). Thus, it is reasonable to postulate that inhibition of NO may abrogate APN-mediated IPo cardioprotection.

Given the role NO plays in cardioprotection, the AdipoR1/Cav-1 signaling pathway which directly increases the production of NO should not be undermined. The knockdown of Cav-1 in endothelial cells significantly blocks APN transmembrane signaling (202). This means that although Cav-1 knockdown may not affect transmembrane signaling in cardiomyocytes, lack of Cav-1 may increase myocardial I/R injury indirectly by impairing blood flow restoration after reperfusion. In the study conducted by Du and colleagues (202), the vasodilatory effect of Cav-1 was further demonstrated by the fact that physiologic APN levels significantly enhanced acetylcholine (Ach)-induced vasorelaxation, an endothelium-dependent and NO mediated process. This effect is abolished when either AdipoR1 or Cav-1 is knocked out. Their study thus supports the notion that the AdipoR1/Cav-1 signaling pathway is essential for APN-mediated NO production, which is essential in the regulation of endothelial function, including vasodilation. The study was done to elucidate the mechanism responsible for physiologically relevant concentrations of APN that induces vasorelaxation, and the potential pathophysiological association with hypertension. However, such a mechanism could contribute to the decrease in blood flow after reperfusion as this would indirectly exacerbate myocardial I/R injury. Thus, therapies that restore the AdipoR1/Cav-1 signaling axis may represent novel modalities for increasing cardioprotection after myocardial I/R injury.

Given that systemic APN malfunction has been identified to be a major risk factor for increased cardiovascular morbidity and mortality in type 2 diabetics, detailed elucidation of the signaling cascade mediated by the AdipoR1/Cav-3 and AdipoR1/Cav-1 interaction would be important. This will not only enhance our understanding of the APN signaling pathway and its regulation, but also provide valuable information on the design of new pharmacological interventions for clinically important diseases such as obesity and type 2 diabetes.

In summary, by virtue of their ability to affect various cellular pathways, both Cav-1 and Cav-3 represent a challenging but interesting therapeutic target for cardioprotection in DCM and in myocardial I/R injury. On the other hand, APN was found to interact with both Cav-1 and Cav-3 in the heart. This suggests APN may serve an important role in the regulation of vasodilation by affecting NO production indirectly *via* its interaction with caveolins. Further, the interaction between Cav-3 and AdipoR1 may be essential for APN-initiated AMPK-dependent anti-oxidative signaling for cardioprotection. Despite the fact that the caveolin scaffolding domain has been proposed as one of the major functional units of caveolins (203, 204), the mechanisms regarding how caveolin scaffolding domain interacts with other signaling proteins such as APN and eNOS as well as the role that the caveolin transmembrane domain (also known as the intramembrane domain) may play in these caveolin-protein interactions remain largely unclear and merit further study. While a few studies have been published on the role of either caveolin or APN in DCM, few studies have examined the interaction between these proteins in the context of DCM or myocardial I/R injury in the functionally impaired diabetic hearts. Hence, APN should be further studied as a pharmacological mediator in the mechanism of action of caveolin-dependent alleviation of the burden of DCM.

## 5 Conclusions

The pathophysiology of DCM is complex and involves relatively unexplored signaling mechanisms, altered substrate preference, and finally structural alterations. Current treatment strategies of DCM still focus on improving glycemic control and enhancing insulin sensitivity, as well as other adjunctive treatments targeting risk factors such as hypertension and hyperlipidemia. Furthermore, treatment of patients with chronic heart failure follows similar principles regardless of whether they have diabetes. Effective treatment options for DCM are lacking. Cav-1/3 and APN are involved in many complex signaling pathways, which creates new opportunities for drug discovery to treat DCM. However, further studies are needed to elucidate the complexity of the caveolin-dependent regulation of APN signaling and features that are clinically applicable.

## Author contributions

Conceptualization: all authors; literature review: all authors; tables: all authors, writing and review: all authors; editing: all authors; revisions and final editing: all authors. All authors contributed to the article and approved the submitted version.

## Funding

The authors’ work was supported by the National Natural Science Foundation of China (No.81970247; 81670770) and by the Hong Kong Research Grant Council (RGC)/GRF (17118619) grant.

## Conflict of interest

The authors declare that the research was conducted in the absence of any commercial or financial relationships that could be construed as a potential conflict of interest.

## Publisher’s note

All claims expressed in this article are solely those of the authors and do not necessarily represent those of their affiliated organizations, or those of the publisher, the editors and the reviewers. Any product that may be evaluated in this article, or claim that may be made by its manufacturer, is not guaranteed or endorsed by the publisher.

## Glossary

**Table:**

DCM	Diabetic Cardiomyopathy
APN	Adiponectin
Cav	Caveolin
T1DM	Type 1 Diabetes Mellitus
T2DM	Type 2 Diabetes Mellitus
FFA	Free Fatty Acid
AGEs	Advanced Glycation End Products
RAAS	Renin-angiotensin-aldosterone System
NO	Nitric Oxide
HFrEF	Heart Failure with Reduced Ejection Fraction
PPARa	Peroxisome Proliferator-activated Receptor alpha
FA	Fatty Acid
ROS	Reactive Oxygen Species
p TNF-a	Tissue Necrosis Factor a
IL	Interleukins
TGF	Transforming Growth Factor
VCAM	Vascular Cell Adhesion Molecule
NF-kB	Nuclear Factor kB
MAPK	Mitogenactivated Protein Kinases
RK	Extracellular signal-regulated Kinases
JUNK	Jun N-terminal Kinases
DAGs	Diacylglycerols
CERs	ceramides
PKC	Protein Kinase C;
ER	Endoplasmic Reticulum
UPR	Unfolded Protein Response’
SERCA	Sarcoplasmic Reticulum Activity
mPTP	mitochondrial Permeability Transition Pore
eNOS	endothelial Nitric Oxide Synthase
O	Nitric Oxide
RAGE	Receptors of Advanced Glycation End Products
I/R	schemia/Reperfusion;
KO – knockout’EC	Excitation-Contraction
Ca	t-tubular Ca2+ current
LTCCs	L-type Ca2+ channels
poE	atherosclerosisprone apolipoprotein E
IR	Insulin Receptor
IRS	Insulin Receptor Substrate
SD	Scaffolding Domain
EHD2	Eps15 omology domain containing pr o t e i n 2
PI3K	Phosphatidylinositol 3-Kinase
PKB	Protein Kinase B
EDHF	Endothelium-derived Hyperpolarizing Factor
GSK	Glycogen Synthase Kinase
1	Heme Oxygenase
LMW	Low Molecular Form
HMW	High Molecular Form
AdipoRs	APN Receptors
AMPK	Adenosine Monophosphate-activated Protein Kinase
LV	Left ventricle
HF	Heart Failure
APPL	Adaptor protein, phosphotyrosine interacting with domain and leucine zipper
MI	Myocardial Ischemia
IPo	Ischemic Postconditioning
MIRI	Myocardial Infarction Reperfusion Injury
Ach	Acetylcholine
